# The complete chloroplast genome of *Chenopodium acuminatum* Willd. (Amaranthaceae)

**DOI:** 10.1080/23802359.2020.1860716

**Published:** 2021-01-16

**Authors:** Hafiz Muhammad Wariss, Xiao-Jian Qu

**Affiliations:** a Cholistan Institute of Desert Studies, The Islamia University of Bahawalpur, Bahawalpur, Pakistan; b Shandong Provincial Key Laboratory of Plant Stress Research, College of Life Sciences, Shandong Normal University, Jinan, China

**Keywords:** *Chenopodium acuminatum*, plastome, phylogenetic analysis

## Abstract

The complete chloroplast genome (plastome) of the *Chenopodium acuminatum* was assembled and annotated in this study. The complete plastome was composed of circular DNA molecules with a total length of 152,200 bp, comprising a large single-copy region (83,683 bp), a small single-copy region (18,131 bp), and two inverted repeat regions (25,193 bp). GC content of this complete plastome was 37.2%. In total, 113 unique genes were annotated, including 79 protein-coding genes (PCGs), 30 transfer RNAs, and 4 ribosomal RNAs. Phylogenomic analysis showed that *C. acuminatum* was closely related to *C. album*.

The genus *Chenopodium* sensu lato, comprising *ca*. 150 species, belongs to the subfamily Chenopodioideae (Amaranthaceae, Caryophyllales), mainly distributed in temperate and subtropical regions of the world (Hong et al. 2017). *Chenopodium* is one of the largest genera in Chenopodioideae subfamily with complex taxonomy due to highly polymorphic leaf shape, floral structure, and seed morphology shared by many *Chenopodium* species (Rahiminejad and Gornall 2004; Kurashige and Agrawal 2005). These are mostly herbaceous, suffrutescent, and arborescent perennial species which were found in arid/semi-arid regions, and saline-alkali soils (Yao et al. 2019). Species of this genus lack typical adaptive structures such as the Kranz type leaf anatomy, the C4 photosynthetic pathway, and succulence when compared to other plants in dry environments (Fuentes-Bazan et al. 2012; Qu, Li, et al. 2019; Zhang et al. 2019). *Chenopodium* species have fascinated many scientists because of their high nutritional value as well source of leafy vegetables worldwide. Furthermore, it contains many bioactive compounds, such as flavonoids, volatile components, saponins and phenolic acids, etc., which is used in herbalism and pharmaceutical industries (Tang and Tsao 2017). *Chenopodium acuminatum* Willd. is an annual herb, mainly distributed in China, Japan, and Korea. *C. acuminatum* is a hermaphrodite plant, pollinated by wind, and generally found along riverbanks, wastelands, and field margins. Plastome has been used to resolve the phylogenetic relationship and development of molecular makers for the identification of taxonomically complex plant species (Jansen et al. 2007; Huang et al. 2020). In this study, we assembled the complete plastome of *C. acuminatum*, in order to facilitate genomic resources to provide insight into systematic and evolution of this important species.

*Chenopodium acuminatum* fresh leaves were collected from Changqing District (Shandong, China; 36°32′N, 116°50′E) and voucher specimen (SD124) was deposited at College of Life Sciences, Shandong Normal University, China. Total genomic DNA was extracted by the modified CTAB method, and was used for library preparation and genome sequencing using Illumina MiSeq at Novogene (Beijing, China). Plastome assembly was conducted by Organelle Genome Assembler (OGA) as described in Qu, Fan, et al. (2019). Plastid Genome Annotator (PGA, (Qu, Moore, et al. 2019)) was used to annotate the complete plastome. The raw sequencing reads was deposited in SRA with the accession number SRR12894192, and the chloroplast genome was deposited in the GenBank with the accession number MW057780.

The complete plastome of *C. acuminatum* was quadripartite structure with 152,200 bp in length, comprising a large single-copy region (83,683bp), a small single-copy region (18,131bp), and a pair of inverted repeat regions (25,193bp). GC content of this complete plastome was 37.2%. A total of 113 unique genes were annotated, including 79 protein-coding genes (PCGs), 30 transfer RNAs, and 4 ribosomal RNAs, among which 13 PCGs and 8 transfer RNAs contained introns.

Phylogenetic relationship of *C. acuminatum* within the Amaranthaceae family was inferred using previously published seventeen plastomes and *Amaranthus caudatus*, *A. hypochondriacus*, *A. hybridus* subsp. *cruentus* and *A. tricolor* were used as outgroups. Maximum-likelihood tree was reconstructed by RAxML v8.2.10 (Stamatakis 2014), based on 79 shared protein-coding genes aligned by MAFFT v7.313 (Katoh and Standley 2013), and with 1000 rapid bootstrap replicates and GTRGAMMA substitution model. Phylogenomic analysis showed that *C. acuminatum* was closely related to *C. album* (Figure 1). This *C. acuminatum* plastome will provide a genomic resources for future systematic studies of this complex genus.

**Figure 1.**
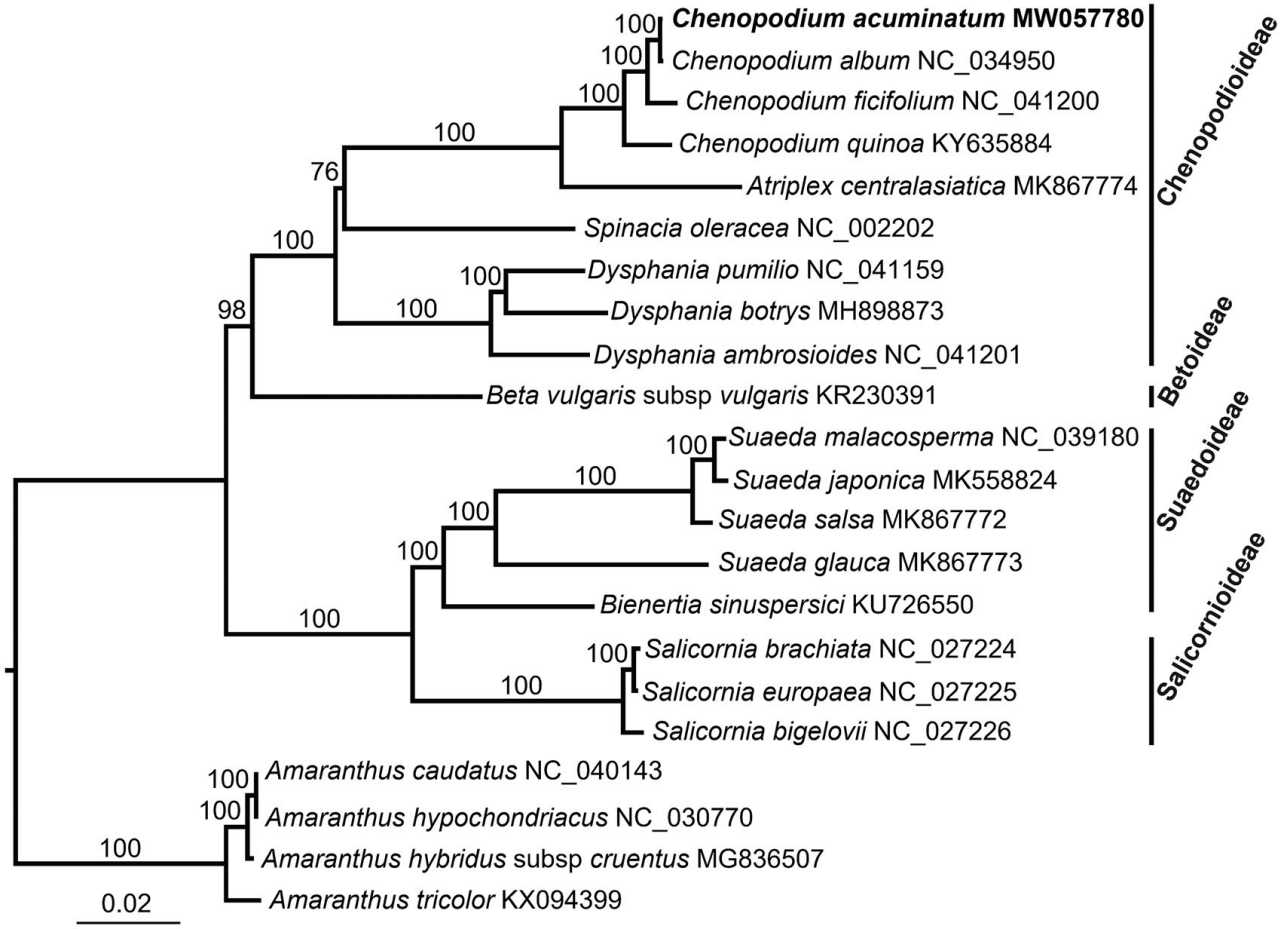
The maximum likelihood tree was reconstructed based on 79 plastome genes. Bootstrap support values are shown on branches. *Chenopodium acuminatum* (MW057780) in this study was indicated by bold font.

## Data Availability

The data that support the findings of this study are openly available in NCBI GenBank at https://www.ncbi.nlm.nih.gov/, SRA accession number SRR12894192 and GenBank accession number MW057780.
